# Superconducting switching due to a triplet component in the Pb/Cu/Ni/Cu/Co_2_Cr_1_*_−x_*Fe*_x_*Al*_y_* spin-valve structure

**DOI:** 10.3762/bjnano.10.144

**Published:** 2019-07-19

**Authors:** Andrey Andreevich Kamashev, Nadir Nurgayazovich Garif’yanov, Aidar Azatovich Validov, Joachim Schumann, Vladislav Kataev, Bernd Büchner, Yakov Victorovich Fominov, Ilgiz Abdulsamatovich Garifullin

**Affiliations:** 1 Zavoisky Physical-Technical Institute, FRC Kazan Scientific Center of RAS, 420029 Kazan, Russia; 2 Leibniz Institute for Solid State and Materials Research IFW Dresden, 01069 Dresden, Germany; 3 Institute for Solid State and Materials Physics, Technical University Dresden, 01062 Dresden, Germany; 4 L. D. Landau Institute for Theoretical Physics RAS, 142432 Chernogolovka, Russia; 5 Moscow Institute of Physics and Technology, 141700 Dolgoprudny, Russia; 6 National Research University Higher School of Economics, 101000 Moscow, Russia

**Keywords:** ferromagnet, proximity effect, superconductor

## Abstract

We report the superconducting properties of the Co_2_Cr_1_*_−x_*Fe*_x_*Al*_y_*/Cu/Ni/Cu/Pb spin-valve structure the magnetic part of which comprises the Heusler alloy layer HA = Co_2_Cr_1_*_−x_*Fe*_x_*Al*_y_* with a high degree of spin polarization (DSP) of the conduction band and a Ni layer of variable thickness. The separation between the superconducting transition curves measured for the parallel (α = 0°) and perpendicular (α = 90°) orientation of the magnetization of the HA and the Ni layers reaches up to 0.5 K (α is the angle between the magnetization of two ferromagnetic layers). For all studied samples the dependence of the superconducting transition temperature *T*_c_ on α demonstrates a deep minimum in the vicinity of the perpendicular configuration of the magnetizations. This suggests that the observed minimum and the corresponding full switching effect of the spin valve is caused by the long-range triplet component of the superconducting condensate in the multilayer. Such a large effect can be attributed to a half-metallic nature of the HA layer, which in the orthogonal configuration efficiently draws off the spin-polarized Cooper pairs from the space between the HA and Ni layers. Our results indicate a significant potential of the concept of a superconducting spin-valve multilayer comprising a half-metallic ferromagnet, recently proposed by A. Singh et al., *Phys. Rev. X ***2015, ***5,* 021019, in achieving large values of the switching effect.

## Introduction

For decades, metallic thin-film heterostructures have been in the in the focus of fundamental research in condensed matter physics and materials science. They show novel fundamental physical phenomena that are absent in the initial materials from which the heterostructures are made. Moreover, those phenomena can also be of remarkable technological relevance. A very prominent example is the discovery, in 1988, of the giant magnetoresistance (GMR) effect in heterostructures composed of alternating layers of ferromagnetic and nonmagnetic metallic layers, which opened a new era of electronics, the so-called spin electronics or, in short, spintronics [1–3].

A new, more recent development in spintronics was based on the idea of integrating superconducting layers into the heterostuctures, which gave rise to the field of superconducting (SC) spintronics (for a review see [4]). The so-called superconducting spin valve (SSV) effect that can be used in SC spintronics was proposed theoretically for the first time by Oh et al. [5] and later on by Tagirov [6]. The construction suggested by Oh et al. was an F1/F2/S structure, where F1 and F2 are the ferromagnetic (F) layers and S is the SC layer, whereas Tagirov proposed a different stacking of the layers F1/S/F2. In both cases the “handle” that switches the SC current in the trilayer is the exchange field from two F layers acting on the S layer. This field is larger for the parallel (P) orientation of the magnetization of the F layers (SC is “off”) rather than for their antiparallel (AP) orientation (SC is “on”). Experimentally, the F1/S/F2 structure was realized first. Gu et al. [7] found in the system CuNi/Nb/CuNi a magnitude of the SSV effect 
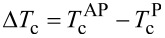
 (where 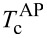 and 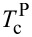 are the SC transition temperatures for the AP and P orientation of the magnetization of the F1 and the F2 layer) of 6 mK and a width of the SC transition curves δ*T*_c_≈ 0.1 K. Unfortunately, the full switching between the normal and SC states could not be achieved because the necessary relation between Δ*T*_c_ and δ*T*_c_, Δ*T*_c_*>* δ*T*_c_ was by far not fulfilled. Since then much experimental work reviewed in [4,8–9] was done until, in 2010, some of the present authors have demonstrated the full on/off switching between the SC and normal states in a Fe1/Cu/Fe2/In heterostructure, as proposed by Oh et al., with Δ*T*_c_ = 19 mK and δ*T*_c_ ≈ 7 mK [10]. That the F1/F2/S structure is indeed beneficial in achieving the full SSV effect was previously indicated by the results in [11], in which possible values of Δ*T*_c_≈ 200 mK in the superlattice [Fe_2_V_11_]_20_ was obtained indirectly.

Another very remarkable advantage of the F1/F2/S system is its functionality as an SC triplet spin valve theoretically predicted by Fominov and co-workers [12]. It is related to the generation of the long-range triplet component (LRTC) of the SC condensate at noncollinear orientations of the magnetization of the F1 and the F2 layer and yields a minimum of the SC critical temperature *T*_c_ of the system in an approximately orthogonal geometry. This theoretical prediction was experimentally confirmed for the first time by some of us in the study of the Fe1/Cu/Fe2/Pb multilayer [13]. A constantly growing experimental and theoretical interest to the various aspects of the LRTC and its implications for the functionality of SSVs has evolved by now into a new area in the field of SC spintronics [4,8–9].

Recently, Singh et al. [14] have reported a record value of 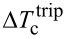 = 0.6–0.8 K due to LRTC in the CrO_2_/Cu/Ni/MoGe heterostructure where one of the F layers was made of the half-metallic compound CrO_2_. 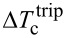 = *T*_c_(α = 0°) − *T*_c_(α = 90°) and α is the angle between the directions of the magnetization of the two F layers. The reason for the large effect was attributed to the efficiency of the half-metallic CrO_2_ layer in drawing off the spin-polarized Cooper pairs from the space between the two F layers.

The goals of the present work were twofold. First, we considered it necessary to verify the breakthrough results by Singh et al. [14], and, second, which was even more important, to answer the question whether the proposed concept of the SSV with a half-metallic F element is of a general character. That is, to find out whether a large SSV effect can be realized using materials other than CrO_2_ in the magnetic part and other than MoGe in the superconducting part of the SSV. Indeed, as will be shown below, we could verify and generalize the results of the pioneering work by Singh and co-workers [14]. Previously, we have shown the advantages of using the Heusler alloy (HA) Co_2_Cr_1_*_−x_*Fe*_x_*Al*_y_* as a weak ferromagnet in the F2 layer of the F1/F2/S SSV structure [15]. Therefore, instead of CrO_2_, which in accordance with the data on point contact spectroscopy [16], has a 90% polarization of the conduction band, we have chosen as a drawing layer for LRTC the HA Co_2_Cr_1_*_−x_*Fe*_x_*Al*_y_* with a spin polarization of the conduction band of ≥70% [17] and instead of MoGe as an S layer we have used the elemental superconductor Pb.

## Sample Preparation and Experimental Results

We prepared several sets of the F1/F2/S spin-valve structures containing HA = Co_2_Cr_1_*_−x_*Fe*_x_*Al*_y_* with the high degree of spin polarization (DSP) of the conduction band standing for the F1 layer adapting the preparation method from [17]. The grown heterostructures have the following composition: MgO/Ta(5 nm)/HA(20 nm)/Cu(4 nm)/Ni(*d*_Ni_)/Cu(1.5 nm)/Pb(105 nm)/Si_3_N_4_ with the variable Ni layer thickness, *d*_Ni_, in the range from 0.6 to 2.5 nm. In this construction MgO(001) is a high-quality single crystalline substrate, Ta(5 nm) is a buffer layer necessary for the optimal growth of the whole structure, HA and Ni play the roles of the ferromagnetic F1 and F2 layer, respectively, Cu(4 nm) decouples the magnetization of the F1 and the F2 layer, Pb(105 nm) is an S layer, Si_3_N_4_ is a protective layer against oxidation, and Cu(1.5 nm) is a buffer layer necessary for the optimal growth of the Pb layer. The Ni, Cu and Pb layers were prepared using e-beam techniques. For the fabrication of the HA and the Si_3_N_4_ layers dc sputtering was used. We used deposition rates of 0.4 Å/s for HA and Si_3_N_4_, 0.5 Å/s for Cu and Ni, and 10 Å/s for Pb. At first, when evaporating HA, the substrate temperature was kept at *T*_sub_ = 700 K to achieve the desired spin polarization of the conduction band of the HA. Indeed, in accordance with our previous work [17] the composition of our alloy, which we call a Heusler alloy, is, in reality, Co_2_Cr_1_*_−x_*Fe*_x_*Al_0.63_ with *x* = 0.48. Obviously, there is deficiency of aluminum in this compound in comparison with the ideal Heusler composition Co_2_Cr_1_*_−x_*Fe*_x_*Al*_y_*. In fact, this “not ideal” composition demonstrates a high DSP of the order of 70% [17]. The study by S. Husain et al. [18] shows that the DSP increases with increasing the substrate temperature *T*_sub_. Therefore, we expect the DSP in our samples to be of the order of 80%. According to our previous study [19], in order to improve the smoothness of the Pb layer the substrate temperature should be reduced down to *T*_sub_≈ 150 K. Therefore, the top Cu(4 nm)/Ni(*d*_Ni_)/Cu(1.5 nm)/Pb fragment was grown at this reduced *T*_sub_. Finally, all samples were covered with a protective Si_3_N_4_ layer to prevent oxidation of the Pb layer.

The Ni layer with the thickness *d*_Ni_≤ 2 nm has coercive field of the order of 2 kOe [20]. In the present study the Ni layer is deposited at a substrate temperature of *T*_sub_≈ 150 K. Therefore its coercive field should be even larger because the density of dislocation increases with decreasing *T*_sub_.

As to the HA layer, our SQUID magnetization measurements show that the onset of the saturation of its magnetization occurs at 30 Oe. At higher magnetic fields the magnetization continues to increase slightly up to the magnetic field of 3 kOe possibly due to some magnetic inhomogeneity of the HA layer. We note that the magnetic response from the Ni layer cannot be resolved here due to its small value.

The electrical resistivity was measured using the standard four-point method. The top insulating layer (Si_3_N_4_) was mechanically removed from the areas where the golden wires should be attached using a silver paste. The quality of the Pb layer can be judged from the residual resistivity ratio (RRR):


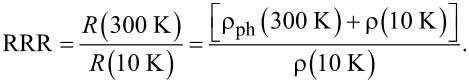


Here, *R*(*T*) is the measured resistance at a given temperature *T*, ρ_ph_(300 K) is the phonon contribution to the specific resistivity at 300 K, and ρ(10 K) is the residual resistivity at 10 K (i.e., above *T*_c_). For our samples this ratio amounted to RRR = 10–12, which corresponds to a SC coherence length of ξ_S_ = 41–45 nm (for details see [21]). The critical temperature *T*_c_ is defined as the midpoint of the SC transition curve. Its width in zero magnetic field varied from 20 to 50 mK depending on the particular series of the samples and increased with the applied field up to ca. 250 mK (see Figure 2 below). The narrow SC transition is a characteristic feature of the high-quality Pb layer.

**Figure 1:**
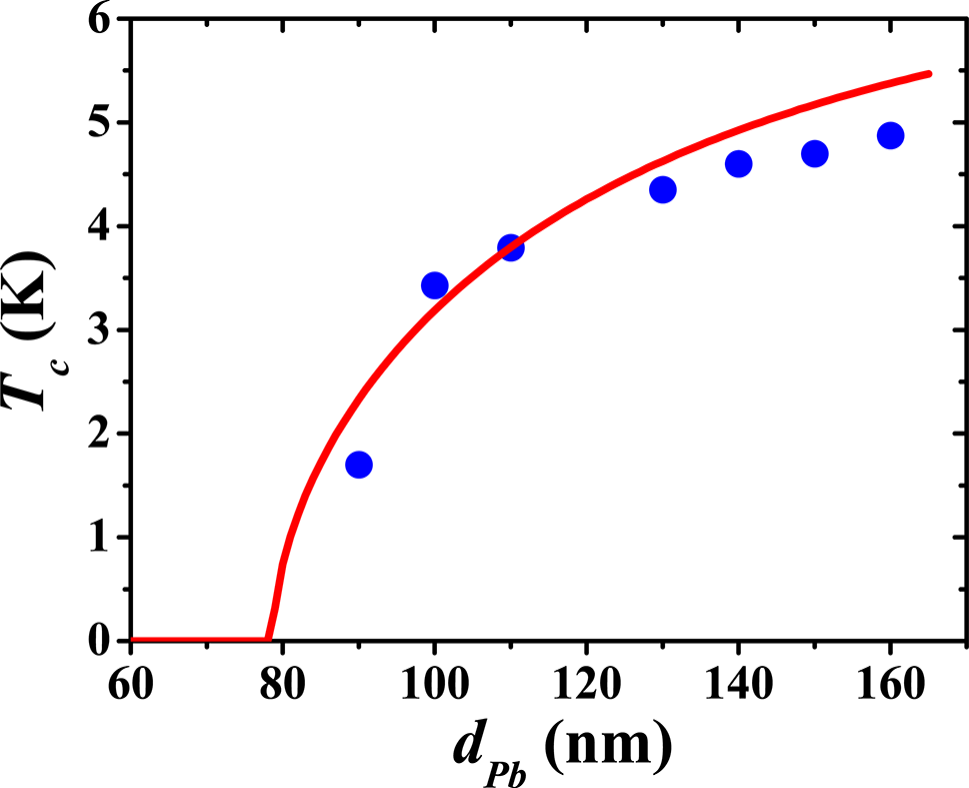
SC critical temperature *T*_c_ as a function of the thickness of the Pb layer *d*_Pb_ at a fixed thickness of the Ni layer *d*_Ni_ = 5 nm for the trilayer Ni(5 nm)/Cu(1.5 nm)/Pb. Solid line is the theoretical fit according to [12] with the coherence length of the Pb layer of ξ_S_ = 41 nm.

For the optimal operation of the SSV it is important to find the optimal thickness of the Pb layer *d*_Pb_. It needs to be sufficiently small to make the whole S layer sensitive to the magnetic part of the system. Only in this case the mutual orientation of the magnetization of the F1 and the F2 layer would affect the *T*_c_ of the stack. In order to determine the optimal thickness we measured the dependence of *T*_c_ on *d*_Pb_ for the heterostructure MgO/Ta(5 nm)/Ni(5 nm)/Cu(1.5 nm)/Pb(*d*_Pb_)/Si_3_N_4_. Figure 1 shows the *T*_c_(*d*_Pb_) dependence for a fixed thickness of the Ni layer *d*_Ni_ = 5 nm, which is much larger than the penetration depth of the Cooper pairs in the Ni layer. The value *T*_c_ decreases rapidly when the thickness of the Pb layer *d*_Pb_ is reduced down to 100 nm. For *d*_Pb_ ≤ 90 nm, values of *T*_c_ ≤ 1.5 K were obtained. Therefore, the optimal thickness range of the Pb layer lies between 90 and 110 nm and for the further study of the SSV effect we have chosen *d*_Pb_ = 105 nm.

Moreover, this procedure is standard for a simple estimation of the boundary parameters. In particular, it enables to determine the critical thickness of the SC layer below which superconductivity vanishes 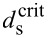. From this we obtain, in accordance with the Appendix in [22], the transparency parameter of the S/F2 interface 
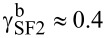
.

For the measurements of the angular dependence of *T*_c_ in the prepared SSV multilayers we have fixed the magnetization of the F2 layer (Ni) in a certain direction by cooling the sample in a magnetic field down to the operational temperatures of the SSV. The magnetization of the F1 layer (HA) can still be easily rotated by an angle α with respect to the pinned magnetization of the Ni layer by an external in-plane field. To manipulate the magnetization direction of the HA layer a magnetic field of 30 Oe is sufficient. We performed such experiments and find a disappointingly small SSV effect. Then, just for curiosity, we extended our study to higher magnetic fields. Surprisingly, we found that with increasing the magnetic field the triplet contribution to the SSV effect linearly increases with the magnetic field. For example, for the sample PLAK4216 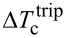 increases linearly up to 0.4 K at 2 kOe (see Figure 4 below).

**Figure 2:**
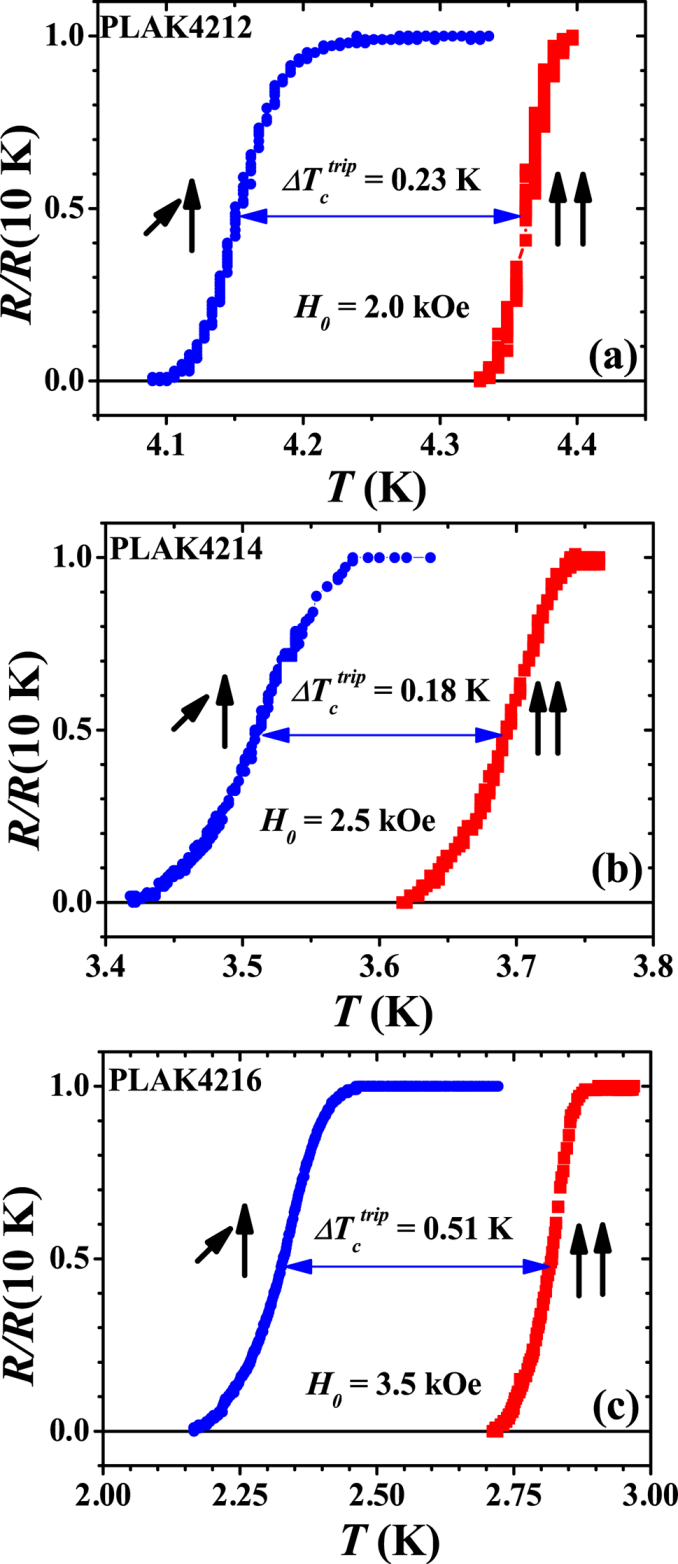
SC transition curves for the P and PP configuration of the cooling field used to fix the direction of the magnetization of the Ni layer and the applied magnetic field *H*_0_, which rotates the magnetization of the HA layer: (a) sample PLAK4212 at *H*_0_ = 2 kOe; (b) sample PLAK4214 at *H*_0_ = 2.5 kOe; (c) sample PLAK4216 at *H*_0_ = 3.5 kOe.

Figure 2 shows the SC transition curves for three representative samples. The shift of the curves between the P (α = 0) and the perpendicular (PP) (α = 90°) orientation, 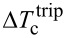, varies between 0.18 and 0.51 K.

**Figure 3:**
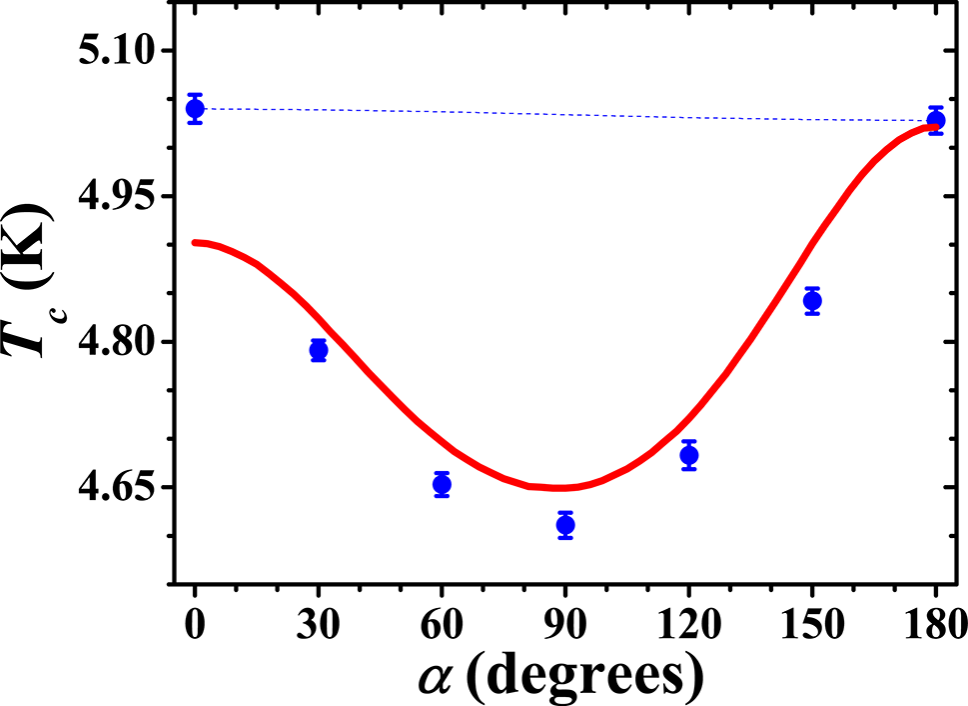
Dependence of *T*_c_ on the angle α between the direction of the cooling field used to fix the direction of the magnetization of the Ni layer and the applied magnetic field *H*_0_ =2 kOe, which rotates the magnetization of the HA layer for sample PLAK4216 HA(20 nm)/Cu(4 nm)/Ni(2.5 nm)/Cu(1.5 nm)/Pb(105 nm). The solid line is the theoretical curve with the parameters presented in section “Discussion”.

Figure 3 depicts the dependence of *T*_c_ on α for the sample PLAK4216. It appears qualitatively similar to the ones observed by us previously ([9,13,15,21,23–26]), reaching a minimum near α = 90°. However, the minimum that we observe now is much deeper, suggesting that the SSV effect is dominated by the spin polarized (triplet) Cooper pairs. The main parameters of the studied SSV samples are listed in Table 1.

**Table 1:** Parameters of all studied samples with variable Ni layer thickness *d*_Ni_. 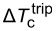 is the maximum value of the triplet SSV effect as determined from the angular dependence *T*_c_(α) obtained at the field *H*_0_.

sample	*d*_Ni_, nm	Δ*T*_c_^trip^, K	*H*_0_, kOe

PLAK4211	0.6	0.05	1.0
PLAK4212	0.9	0.23	2.0
PLAK4213	1.3	0.13	2.0
PLAK4214	1.6	0.18	2.5
PLAK4215	2	0.05	1.25
PLAK4216	2.5	0.51	3.5

The magnitude of the triplet SSV effect 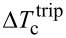 depends practically linearly on *H* at small applied magnetic fields up to the field *H*_0_ the values of which are listed in Table 1 together with the corresponding values of 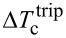.

**Figure 4:**
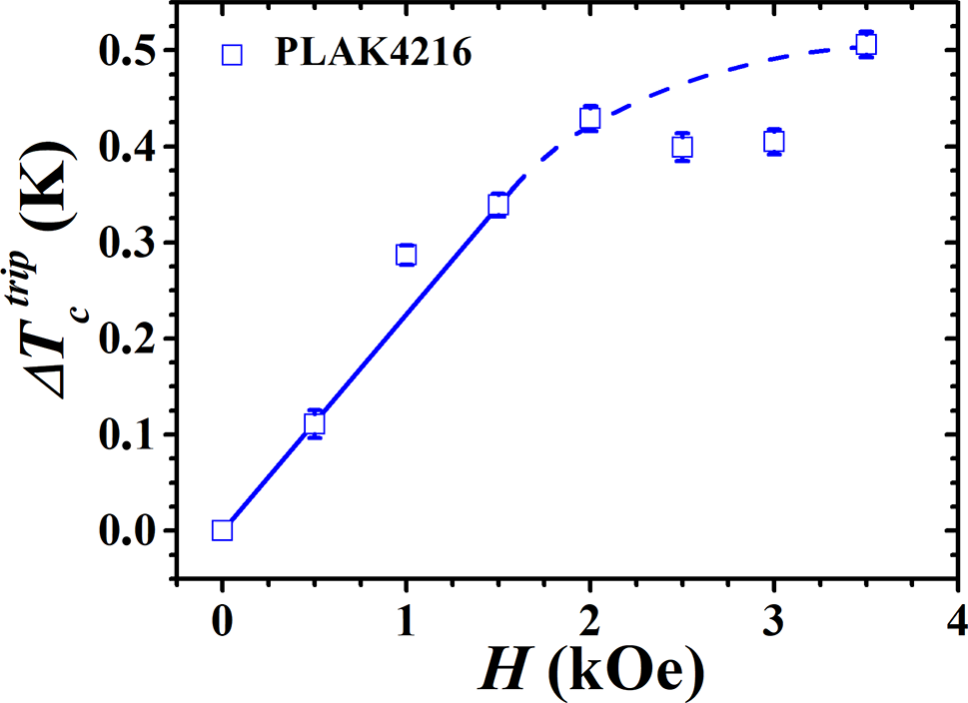
The magnitude of the triplet SSV as a function of the applied magnetic field for sample PLAK4216. The line is a guide to the eyes.

At first glance it is surprising that 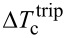 increases well above the saturation magnetic field for the HA layer. We suppose this may be caused by some magnetic inhomogeneity of the HA layer reflected in a slight increase of its magnetization up to the field of 3 kOe, where more and more “microdomains” become gradually involved in the formation of the total moment. Figure 4 shows the dependence of the triplet contribution to the SSV effect on the external magnetic field for the sample PLAK4216. A similar increase of 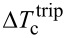 was observed by Singh et al. [14], for which no conclusive explanation can be found at present. This field-dependent effect observed by two groups on different samples appears to be a salient feature of the new type of SSVs and needs theoretical explanation.

## Discussion

A remarkably large separation of the SC transition curves for the P and the PP orientation of the magnetization of the F1 and the F2 layer yielding values of 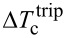 up to 0.5 K in not too strong magnetic fields compared to those in [14] evidences prominent spin–triplet superconducting correlations in our samples. The theoretical approach that we employ for the analysis of the experiment in Figure 3 is based on a generalization of [12] along the lines of [27–28]. It allows to consider layered structures with different material parameters of all the layers and arbitrary Kupriyanov–Lukichev boundary parameters [29] of all the interfaces.

Each of the two interfaces (F/S and F/F) is described by the matching parameter γ and the resistance parameter γ_b_ [27]:

[1]
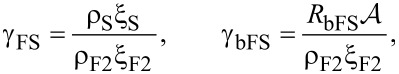


[2]
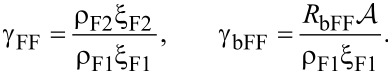


Here, ρ and ξ are the resistance and the coherence length of the layers, *R*_b_ and 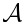 are the interface resistance and area. The necessity to consider arbitrary F/F interface parameters is due to different materials of the two F layers. This is a new theoretical ingredient, in comparison to fittings of our previous experiments in [9,13,21]. Figure 3 demonstrates that theory correctly reproduces characteristic features of the *T*_c_(α) dependence (triplet spin-valve behavior). Parameters used for fitting of the theory to the experimental results are the following: coherence length in the S-layer, ξ_S_ = 41 nm, coherence lengths in the F2 and the F1-layer, ξ_F2_ = 6.25 nm and ξ_F1_ = 40 nm. The boundary conditions of the S/F and F/F interfaces are given by the material matching parameter γ and the transparency parameter γ^b^, γ_SF_ = 0.1, 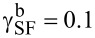, γ_FF_ = 1, and 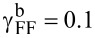. The exchange energy of the F1 and of the F2 layer is *h*_2_ = 0.03 eV and *h*_1_ = 0.39 eV, respectively.

## Conclusion

When studying the SSV multilayers Co_2_Cr_1_*_−x_*Fe*_x_*Al*_y_*/Cu/Ni/Cu/Pb the magnetic part of which contains the Heusler alloy Co_2_Cr_1_*_−x_*Fe*_x_*Al*_y_* with a high degree of spin polarization of the conduction band we have obtained a large SSV effect due to the long-range triplet component of the superconducting condensate 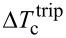 ≈ 0.5 K at a moderate applied field of 3.5 kOe as compared with the earlier work in [14]. Our results show that there is a potential to achieve large values of 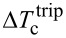 even at smaller fields relevant for applications by careful design and optimization of all elements of the SSV heterostructure regarding both the superconducting and the magnetic part. Our observations suggest that the concept of a SSV with a half-metallic ferromagnetic element proposed in [14] is of general character. In particular, finding for this purpose the most appropriate ferromagnet with a high degree of spin polarization of the conduction band appears to be a crucial issue. Furthermore, noting first theoretical attempts in [30–31], our data as well as the results by Singh et al. [14] call for a comprehensive quantitative theoretical treatment to obtain further insights into exciting physics of the triplet superconducting spin valves.
